# Novel Mechanism and Kinetics of Tetramethrin Degradation Using an Indigenous *Gordonia cholesterolivorans* A16

**DOI:** 10.3390/ijms22179242

**Published:** 2021-08-26

**Authors:** Yuxin Guo, Yaohua Huang, Shimei Pang, Tianhao Zhou, Ziqiu Lin, Hongxiao Yu, Guorui Zhang, Pankaj Bhatt, Shaohua Chen

**Affiliations:** 1 Key Laboratory for Conservation and Utilization of Subtropical Agro-Bioresources, Integrative Microbiology Research Centre, South China Agricultural University, Guangzhou 510642, China; yuxinguo@stu.scau.edu.cn (Y.G.); 20183138021@stu.scau.edu.cn (Y.H.); 20192047012@stu.scau.edu.cn (S.P.); melo.dn07@gmail.com (T.Z.); 20192047010@stu.scau.edu.cn (Z.L.); hongxiaoyu@stu.scau.edu.cn (H.Y.); 201813070127@stu.scau.edu.cn (G.Z.); 2 Guangdong Laboratory for Lingnan Modern Agriculture, Guangzhou 510642, China

**Keywords:** tetramethrin, *Gordonia cholesterolivorans*, bioaugmentation, metabolites, metabolic pathways

## Abstract

Tetramethrin is a pyrethroid insecticide that is commonly used worldwide. The toxicity of this insecticide into the living system is an important concern. In this study, a novel tetramethrin-degrading bacterial strain named A16 was isolated from the activated sludge and identified as *Gordonia cholesterolivorans*. Strain A16 exhibited superior tetramethrin degradation activity, and utilized tetramethrin as the sole carbon source for growth in a mineral salt medium (MSM). High-performance liquid chromatography (HPLC) analysis revealed that the A16 strain was able to completely degrade 25 mg·L^−1^ of tetramethrin after 9 days of incubation. Strain A16 effectively degraded tetramethrin at temperature 20–40 °C, pH 5–9, and initial tetramethrin 25–800 mg·L^−1^. The maximum specific degradation rate (*q*_max_), half-saturation constant (*K*_s_), and inhibition constant (*K*_i_) were determined to be 0.4561 day^−1^, 7.3 mg·L^−1^, and 75.2 mg·L^−1^, respectively. The Box–Behnken design was used to optimize degradation conditions, and maximum degradation was observed at pH 8.5 and a temperature of 38 °C. Five intermediate metabolites were identified after analyzing the degradation products through gas chromatography–mass spectrometry (GC-MS), which suggested that tetramethrin could be degraded first by cleavage of its carboxylester bond, followed by degradation of the five-carbon ring and its subsequent metabolism. This is the first report of a metabolic pathway of tetramethrin in a microorganism. Furthermore, bioaugmentation of tetramethrin-contaminated soils (50 mg·kg^−1^) with strain A16 (1.0 × 10^7^ cells g^−1^ of soil) significantly accelerated the degradation rate of tetramethrin, and 74.1% and 82.9% of tetramethrin was removed from sterile and non-sterile soils within 11 days, respectively. The strain A16 was also capable of efficiently degrading a broad spectrum of synthetic pyrethroids including D-cyphenothrin, chlorempenthrin, prallethrin, and allethrin, with a degradation efficiency of 68.3%, 60.7%, 91.6%, and 94.7%, respectively, after being cultured under the same conditions for 11 days. The results of the present study confirmed the bioremediation potential of strain A16 from a contaminated environment.

## 1. Introduction

Natural pyrethrin insecticides were produced by *Tanacetum cinerariifolium* [1]. Natural pyrethrins were used in the early prevention and control of domestic health pests [2]. Their mechanism of action is to interfere with the nerve sodium ion channel and block the nerve signal transmission [3]. Synthetic pyrethroids derived from natural pyrethrins have light and heat stability, which provide an alternative to widely used organophosphorus and organochlorine pesticides that pose a threat to human health and the environment [4]. The synthetic pyrethroids can be divided into type I pyrethroids, which lack a cyano group, and type II pyrethroids, which have a cyano group [5]. The chemical structure of pyrethroids contains chiral centers. Due to their high activity and low residue, they are still one of the pillars in the field of pesticides. The use of pyrethroid insecticides is increasing annually, from representing 25% of the global pesticide market in 2010 to more than 30% in 2018 [6].

However, pyrethroid insecticides also have some side effects on the environment. Previous studies on their metabolic pathways and hazards in the environment have mostly focused on soil. With the widespread use of pyrethroid insecticides, they can enter the natural water cycle through surface water flow, infiltration through soil, and via other routes, causing pollution to domestic water and riverbed sediments, and leaving serious indoor residues [7]. As a synthetic chemical pesticide, excessive use of pyrethroid insecticides or non-standard operations will inevitably lead to them entering the soil or natural water cycle, with negative effects on plants and animals. Pyrethroids are highly hydrophobic and are quickly adsorbed into sediments when they enter water [8]. Studies have shown that pesticides of pyrethroids can induce changes in the erythrocyte membrane in rats, resulting in a significant increase in erythrocyte membrane phospholipids and a decrease in cholesterol levels [9]. At the same time, deltamethrin significantly inhibited the hatching rate of carp at a concentration of 50 μg·L^−1^, with a hatching rate of only 9% [10]. As a chiral compound, pyrethroids usually have 4–8 stereoisomers [11]. Different isomers exhibit different insecticidal activities and selective degradation characteristics during microbial metabolism [12]. For example, αS-2S-fenvalerate can cause obvious bodily distortion and pericardial cysts in zebrafish, and the 96 h mortality of zebrafish induced by αS-2S-fenvalerate was 3.8 times higher than that of other enantiomers [13]. Previous research has shown that pyrethroids are not acutely toxic to the human body, but long-term exposure to low doses of the environment can damage sperm DNA, affecting sperm quality. For example, biphenyl chrysanthemum ester reduced the sperm mRNA expression of survival [14].

Environmental problems caused by the large-scale use of pyrethroid insecticides have become increasingly serious. Tetramethrin (2,2-dimethyl-3-(2-methyl-1-propenyl)cyclopropanecarboxylic acid (1,3,4,5,6,7-hexahydro-1,3-dioxo-2*H*-isoindol-2-yl)methyl ester) is a popular type I pyrethroid. The continuous use of tetramethrin and its environmental residues have caused serious concern. Therefore, it is urgent to develop effective strategies to solve the problems related to tetramethrin residues. Microbial degradation of organic pollutants in the environment has attracted widespread attention [15]. Compared with physical and chemical methods, biodegradation is more effective and safe, has lower costs, and causes no secondary pollution [16]. However, at the bacterial level, microbial degradation also poses some problems because bacterial passivation cannot achieve effective degradation [17]. Using the abundant microbial resources to isolate and screen efficient pyrethroid-degrading bacteria, and to establish a germplasm resource library of specific pesticide-degrading bacteria is of great significance for the control of pesticide residues and environmental remediation [18]. To date, several studies have demonstrated that bacteria and fungi can effectively degrade pyrethroid residues in water and soil environments. Microorganisms can degrade pyrethroids through co-metabolism, using pyrethroids as their sole energy source or with the assistance of other nutrients [19]. The final product of pyrethroid degradation is aliphatic short-chain hydrocarbons, which are easily used by microorganisms through their universal metabolic pathways [20]. These microbial strains include *Bacillus subtilis* 1D, *Photobacterium ganghwense* 6046, *Acinetobacter baumannii* ZH-14, and *Paracoccus acridae* SCU-M53, among others [21,22,23,24]. At present, research on tetramethrin mainly focuses on its toxicity and residue analysis, while there are very few studies that have reported on the biodegradation of tetramethrin, its degradation mechanism, and the possible metabolic pathways involved. Due to the scarce information in the literature concerning tetramethrin degradation when compared with other pyrethroids, microbial degradation should be investigated to improve the scientific understanding and environmental fate of this insecticide.

In this study, the role of strain A16 was investigated for the biodegradation of tetramethrin with the following objectives: (i) to isolate and characterize the tetramethrin-degrading strain from activated sludge, (ii) to optimize the process parameters for tetramethrin degradation using response surface methodology, (iii) to test the degradation kinetics of tetramethrin and other pyrethroids, (iv) to identify the intermediate metabolites formed during tetramethrin degradation, and (v) to estimate the bioremediation potential of strain A16 in water and soil environments.

## 2. Results and Discussion

### 2.1. Isolation, Enrichment, and Characterization of the Bacterial Strain A16

**Figure 1:**
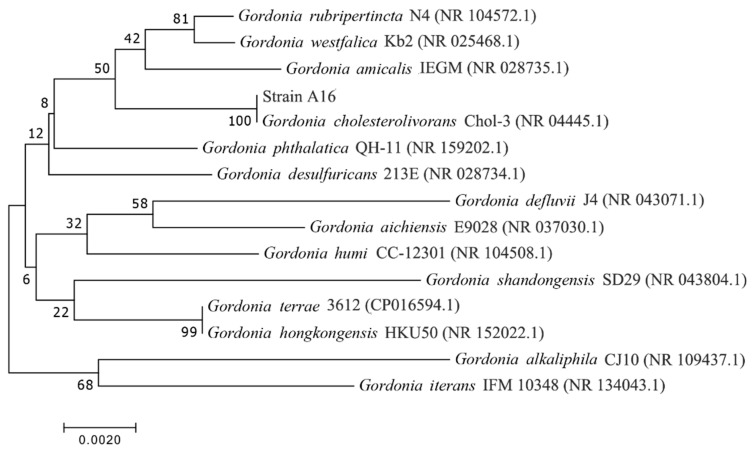
Phylogenetic analysis based on 16S rRNA sequence of strain A16 and similar bacterial strains. The phylogenetic tree was constructed using the neighbor-joining (NJ) method. Numbers in parentheses represent the GenBank accession numbers of the sequences.

A bacterial strain that was able to degrade the tetramethrin and other pyrethroids was isolated and characterized using physical, chemical, and microbiological methods. The potential strain was designated as A16. Strain A16 not only degraded the tetramethrin, but was also found to effectively degrade other pyrethroids such as prallethrin, fenvalerate, bifenthrin, beta-cypermethrin, allethrin, and permethrin. The colonies of the degrading strain A16 on beef extract peptone solid medium were white and yellow, and the single colonies were circular. The color from the middle to the edge became lighter, and its edges were irregular. The colony surface was translucent. Strain A16 cells were rod-shaped, without spores and flagella (Figure S1). The genomic DNA of strain A16 was used as a template for PCR amplification by a universal set of primers, and the amplified products were sequenced. Furthermore, the sequence gene was deposited in GenBank with accession number MW872315. The phylogenetic analysis revealed that strain A16 showed 99% similarity with *Gordonia cholesterolivorans*. Thus, strain A16 was identified as *G. cholesterolivorans* strain A16. The phylogenetic tree of strain A16 is represented in Figure 1.

Previous researchers reported that the *Gordonia* species showed potential to degrade other organic pollutants [25,26,27]. However, *Gordonella* has not been reported to degrade pyrethroid pesticides, so there is still room for exploration and development of the biodegradation ability of pyrethroid pesticides by *Gordonella*. In addition, *Bacillus*, *Pseudomonas*, *Raoultella*, *Brevibacterium*, *Aspergillus*, *Acinetobacter*, *Candida,* and *Trichoderma* have been characterized on the basis of biochemical and molecular tools for their pyrethroid degradation potential in soil and water environments [28,29,30,31,32,33,34,35]. Our study provides the first evidence that *Gordonella* bacterial strains participate in highly effective degradation of tetramethrin and other pyrethroids.

### 2.2. Growth Linked Degradation of Tetramethrin with Strain A16

**Figure 2:**
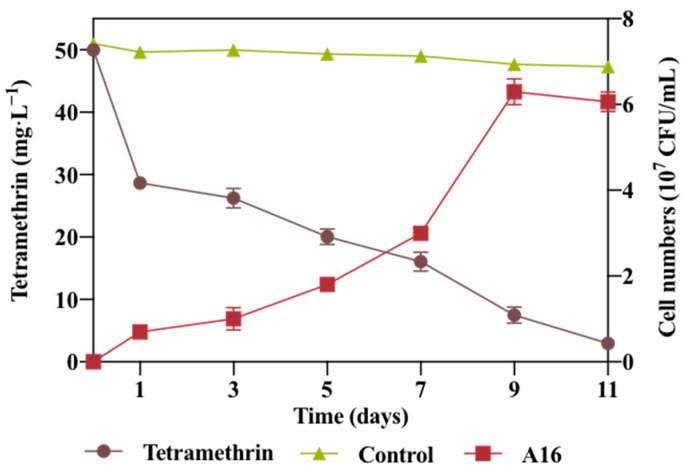
Growth-linked degradation of tetramethrin in mineral salt medium (MSM) by strain A16.

The growth-linked degradation study clearly showed that as the growth of bacterial strain A16 increased over time, the overall concentration of tetramethrin present in the medium decreased (Figure 2). Compared with the control, strain A16 enhanced the degradation rate of tetramethrin. The rate of degradation for tetramethrin was affected according to the microbial growth curve. As the microbial density reached the logarithmic phase, the maximum rate of degradation was achieved, while it decreased once the microbial growth curve reached the static phase. After 7 and 11 days of incubation, the tetramethrin was degraded by over 70% and 95%, respectively. It can be assumed that strain A16 can utilize tetramethrin as a sole source of carbon for growth, revealing its promising potential for the bioremediation of a tetramethrin-contaminated environment. Furthermore, strain A16 effectively degraded other pyrethroids, suggesting that the bacterial enzymes involved in the degradation process were not substrate specific.

A growth-linked biodegradation study was also conducted previously and reported that bacterial strain *Bacillus* sp. FA4 degraded fipronil pesticide (50 mg·L^−^^1^), as the bacterial cell density increased in the pesticide supplemented medium under optimized conditions [36]. In a mineral medium the bacterial strain utilized the supplemented material as its sole source of carbon/nitrogen or energy to sustain its growth and metabolism [37]. The mechanism of pesticide biodegradation is of great interest to researchers and it depends on different factors such as the genetic pool of microbial communities, co-metabolism, electron donors (glucose, sucrose, acetic acid, pyruvic acid) that induce the biotransformation process, and the activation or induction of pesticide-degrading genes that enhance degradation rates under stress conditions [38]. Pesticides that support the growth of microorganisms could be subjected to pesticide biodegradation [39].

### 2.3. Degradation Kinetics of Strain A16 with Different Initial Concentrations of Tetramethrin

**Figure 3:**
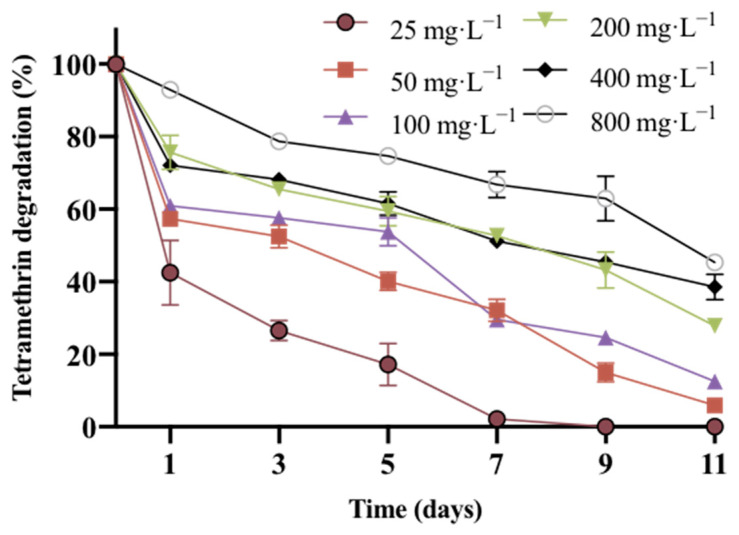
Degradation ability of strain A16 to tetramethrin at different concentrations.

The degradation kinetics of different concentrations of tetramethrin (25–800 mg·L^−1^) by strain A16 showed that it can tolerate high concentrations and rapidly degrade tetramethrin. As shown in Figure 3, after 11 days of incubation in MSM medium with 25 mg·L^−1^, 50 mg·L^−1^, 100 mg·L^−1^, 200 mg·L^−1^, 400 mg·L^−1^, and 800 mg·L^−1^ of tetramethrin, the degradation efficiency was 100%, 95.1%, 89.6%, 72.6%, 65.0%, and 56.7%, respectively. When the concentration of tetramethrin was 25 mg·L^−1^, the degradation efficiency reached 95.7% after 7 days of incubation, and it was completely degraded after 9 days of incubation. The results showed that strain A16 had a good degradation effect at low concentrations (<100 mg·L^−1^). However, with the increase of the concentration of tetramethrin, the degradation rate decreased, which might be due to the strong toxicity of high concentrations of tetramethrin.

**Figure 4:**
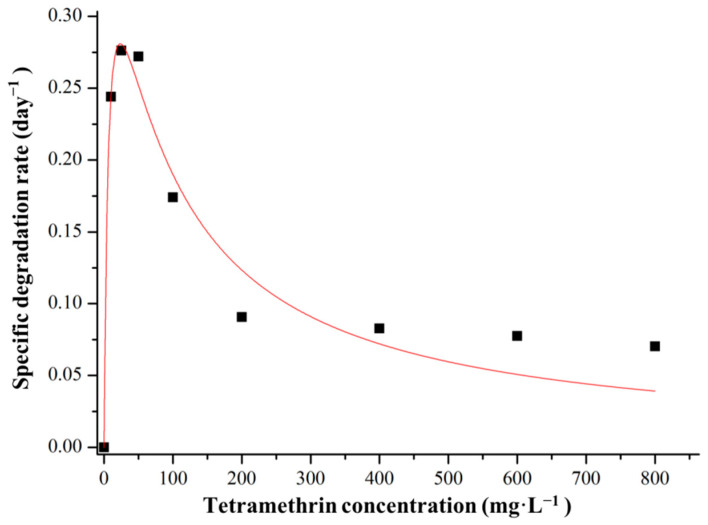
Relationship between the initial tetramethrin concentration and specific degradation rate using strain A16.

The results showed that the degradation efficiency of strain A16 decreased with the increase of the concentration of tetramethrin, indicating that tetramethrin could be used as a substrate for the growth of strain A16 during the biodegradation process. At high concentrations, it could also inhibit the growth of strain A16. Thus, the Andrews equation was used to simulate the kinetic analysis of the biodegradation process of tetramethrin at different concentrations (Figure 4). The degradation kinetic results showed that the theoretical value of the model was in good agreement with the actual value (*R*^2^ = 0.9424), and the maximum specific degradation rate (*q*_max_) of tetramethrin was 0.4561 day^−1^. The half rate constant (*K*_s_) was 7.3 mg·L^−1^ and the inhibition coefficient (*K*_i_) was 75.2 mg·L^−1^. The Andrews equation was then further derived, and the corresponding concentration (*S*_max_) of *q*_max_ was 23.5 mg·L^−1^, which was the best predicted concentration for the degradation of tetramethrin by strain A16.

Enzymatic kinetics (parameters were *K*_i_ = 479.4 mg·L^−1^; *K*_s_ = 12.1 mg·L^−1^; *q*_max_ = 0.6980 day^−1^) of fipronil degradation in a mineral salt medium with bacterial strain *Bacillus* sp. FA3 were performed, which significantly reduced the half-life of fipronil [36,40]. The comparative study of pesticide degradation in sterilized soil and an immobilized bacterial cell with sodium alginate beads and agar disk demonstrated that the half-life of pesticide reduced rapidly (7.8 and 7.3 days) in an immobilized medium when compared with sterilized soil (169.0 days) [36,40]. This increased rate of degradation may be due to the production of an organic intermediate that can act as an electron donor, which helps reduce the half-life of the compound significantly.

### 2.4. Optimization of Tetramethrin Degradation Conditions

The Box–Behnken design based on RSM was used to optimize the degradation conditions of tetramethrin in strain A16. Based on the preliminary study of single factor experiment, three independent variables were selected—temperature (*X*_1_), pH (*X*_2_), and inoculation amount (*X*_3_)—to predict the optimal degradation parameters for strain A16. The residues of tetramethrin (*Y*_1_) obtained by HPLC analysis represented the effects of three variables on strain A16 at different levels. Table 1 lists the experimental design variables corresponding to the degradation of tetramethrin. A polynomial regression analysis was carried out on the relevant degradation data through response surface, and the experimental values of degradation of tetramethrin were fitted to obtain the following quadratic binomial regression equation (Equation (1)):

*Y* = 98.28 + 1.76*X*_1_ + 14.94*X*_2_ + 5.06*X*_3_ + 7.68*X*_1_*X*_2_ + 4.00*X*_1_*X*_3_ + 10.66*X*_2_*X*_3_ − 7.22*X*_1_^2^ − 38.27*X*_2_^2^ + 0.7213*X*_3_^2^ (1)

where *Y*_1_ is the predicted tetramethrin degradation (%); and *X*_1_, *X*_2_, and *X*_3_ are coded values of temperature, pH, and inoculum size, respectively.

The analysis of variance (ANOVA) of the quadratic polynomial model is presented in Table 2. The *F* value of the model term was 15.3 and the *p*-value < 0.05, indicating that the equation significantly fitted the degradation process of tetramethrin. Statistical analysis results show that the determination coefficient *R*^2^ was 0.9650, indicating that the actual value of this experiment and the predicted value of the model have a high degree of agreement. A low coefficient of variation (CV = 1.7%) suggested that the model was accurate and reliable. The regression analysis showed that the single factor and square terms of temperature (*X*_1_) and pH (*X*_2_), namely *X*_1_, *X*_2_, *X*_1_^2^ and *X*_2_^2^, had significant effects on the degradation of tetramethrin by strain A16 (*p* < 0.05), while *X*_3_^2^ had no significant effects on the degradation of tetramethrin by strain A16 (*p* > 0.05). The interaction term of inoculation size (*X*_3_) and pH (*X*_2_) had no significant effect on the metabolic activity of strain A16 (*p* > 0.05).

**Figure 5:**
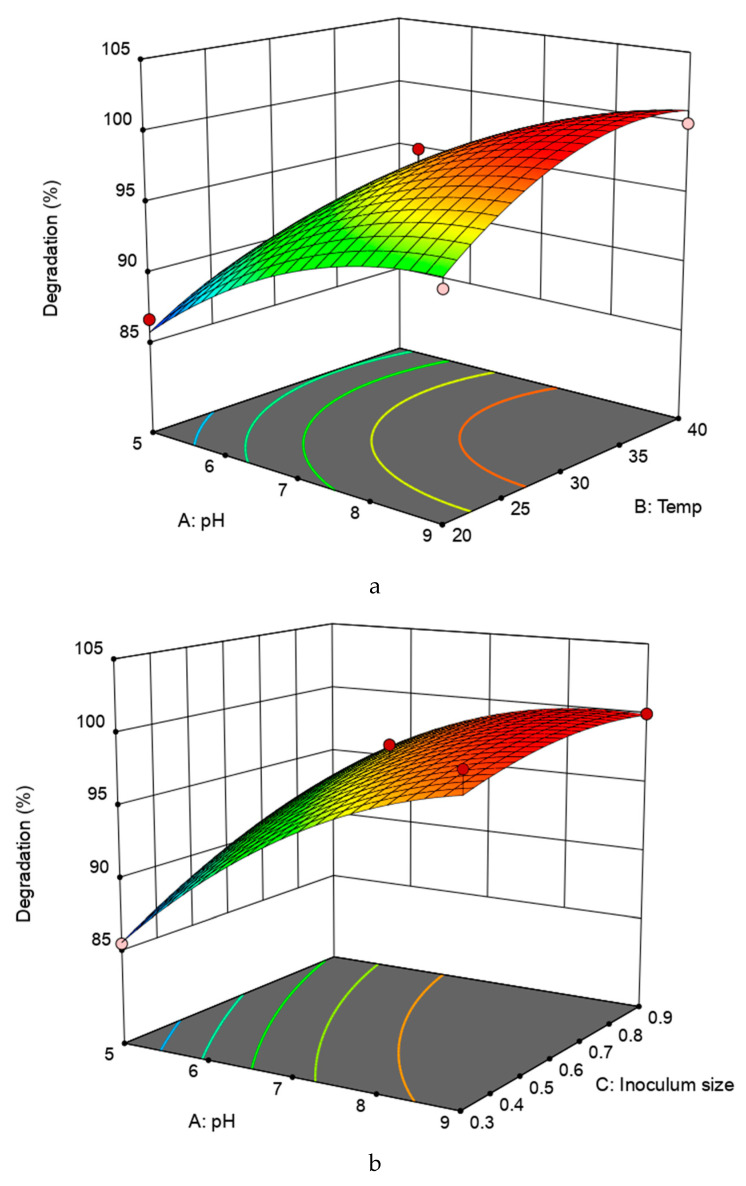

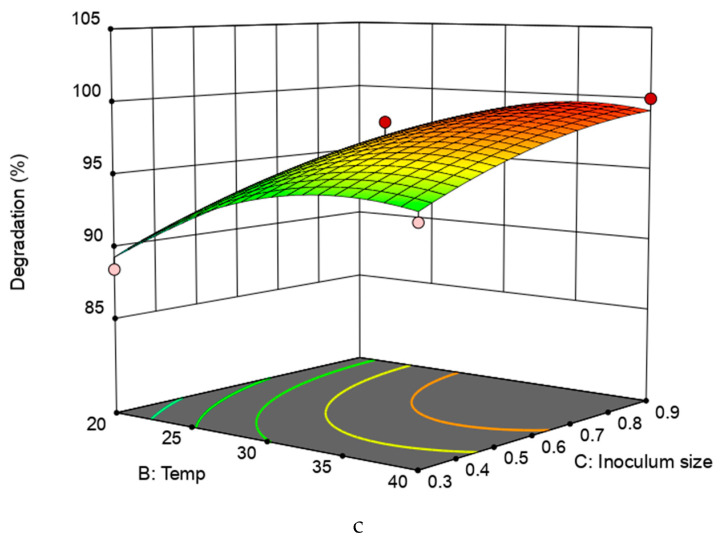
Response surface showing the interactive effects of pH (A), temperature (B), and inoculum size (C) on tetramethrin degradation by strain A16. (**a**) 3D plot illustrating the interactive effect of pH (A), temperature (B) on tetramethrin degradation; (**b**) 3D plot illustrating the interactive effect of pH (A) and inoculum size (C) on tetramethrin degradation; (**c**) 3D plot illustrating the interactive effect of temperature (B) and inoculum size (C) on tetramethrin degradation.

To improve our understanding of the results, we produced a three-dimensional response surface which illustrates the effects of temperature (*X*_1_), pH (*X*_2_), and inoculum size (*X*_3_) on tetramethrin degradation with different independent variables as a constant that indicate the maximum degradation that could be achieved inside the design boundaries (Figure 5). The model depicts a maximum degradation of 100% at temperature 38.3 °C pH 8.5, and an OD value of inoculum size of 0.8.

Strain A16 was capable of rapidly degrading tetramethrin without a lag phase over a wide range of temperatures (20–40 °C) and pH values (5–9). This is an important feature of bioremediation by organisms in a variable environment [41]. The results show that degradation occurs more easily under alkaline conditions, which is consistent with the findings of previous studies [42,43,44]. In addition, the acidic conditions reduced the activity of strain A16, resulting in weak degradation of tetramethrin. The Box–Behnken design based on RSM methods have been implemented well in pyrethroid degradation performed by previous researchers [21]. The optimization of the pH, temperature, concentration, and inoculum size successfully predicted the degradation of cypermethrin, allethrin, and cyhalothrin [45,46,47]. Recently, *Bacillus thuringiensis* Berliner involved in degradation of cypermethrin was investigated and the degradation parameters were optimized using RSM. This group of researchers implemented a central composite design for the optimization of the critical parameters of concentration, pH, and temperature in relation to cypermethrin degradation [48].

### 2.5. Degradation of Various Synthetic Pyrethroids by Strain A16

**Figure 6:**
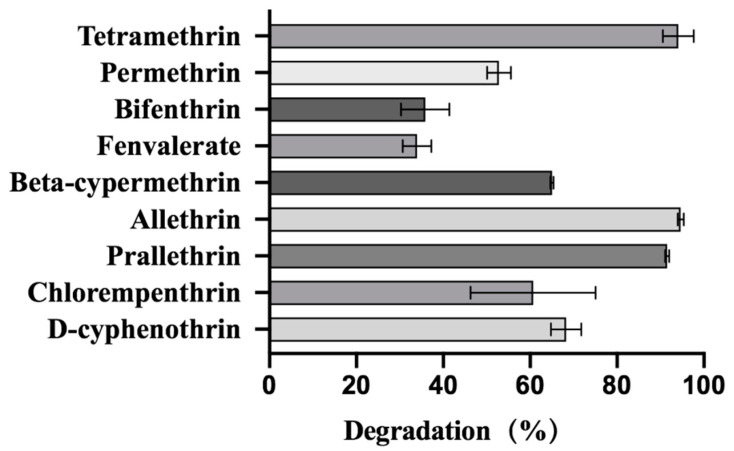
Degradation of various synthetic pyrethroid insecticides by strain A16.

Our results showed effective degradation with various pyrethroids by strain A16 (Figure 6). The degradation effects of strain A16 on different synthetic pyrethroids were significantly different. After 11 days of treatment under the same conditions, the degradation efficiency of strain A16 on permethrin, chlorempenthrin, beta-cypermethrin, D-cyphenothrin, prallethrin, allethrin, and tetramethrin reached 52.8%, 60.7%, 65.0%, 68.3%, 91.6%, 94.7%, and 95.1%, respectively. Strain A16 showed greater degradation effects on most synthetic pyrethroids, while its degradation effect against fenvalerate and bifenthrin was only 33.9% and 35.8%, respectively.

Previous studies showed that the degradation activity of pyrethroid-degrading strains was significantly inhibited at high pyrethroid concentrations [49,50,51]. This particular strain was found to be highly effective in degrading tetramethrin up to the concentration of 800 mg·L^−1^. Meanwhile, strain A16 showed no obvious substrate specificity for various typical pyrethroid pesticides, suggesting that strain A16 may be suitable for bioremediation of various polluted environments. A single microbial strain can degrade many pyrethroids through its wider metabolism [52,53,54]. *Bacillus subtilis* BSF01 has been investigated for its degradation of cypermethrin, cyfluthrin and cyhalothrin, and broader substrate specificity was reported due to the expression of the carboxylesterase CesB [55].

### 2.6. Degradation Kinetics of Tetramethrin in Soil Slurry

**Figure 7:**
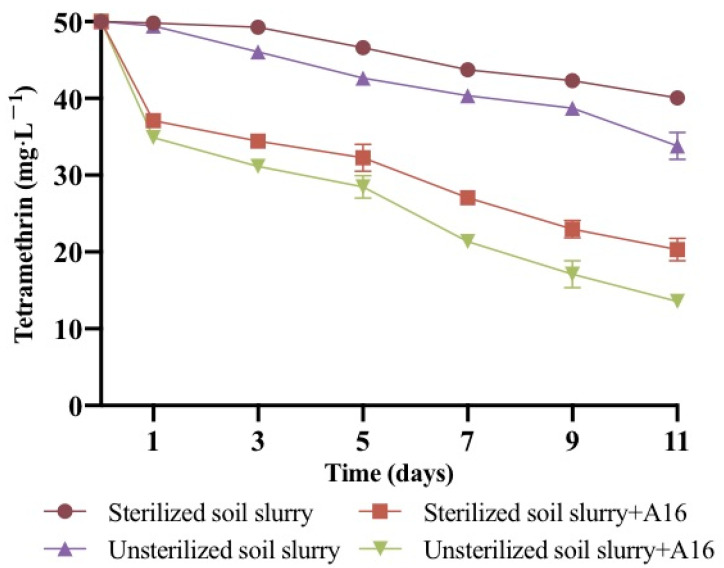
Degradation kinetics of tetramethrin in different soil slurries.

This experiment simulated the degradation ability of strain A16 in soil slurry and the results are shown in Figure 7. Soil slurry biodegradation experiments were carried out under sterile and non-sterile conditions, while a non-inoculated treatment was used as the control.

The degradation efficiency of strain A16 for tetramethrin (50 mg·L^−^^1^) in unsterilized soil-containing slurry reached 82.9%, while for tetramethrin sterilized slurry it reached 74.1% after 11 days of experiment. The results showed that there was no non-degradation or delayed degradation by strain A16 after the sludge was inoculated, and the degradation ability in the unsterilized soil slurry was always higher than in the sterilized soil slurry. Similarly, the degradation efficiency of tetramethrin in unsterilized soil slurry was higher than in sterilized soil slurry when strain A16 was not added in this experiment, suggesting that the indigenous soil microflora play a role in the degradation of tetramethrin. The degradation process of tetramethrin in soil slurry followed the first-order kinetics equation. The degradation constant (*k*) of tetramethrin with strain A16 was 0.0048 and 0.0054 day^−1^ in sterilized and unsterilized soil slurry treatment groups, respectively, whereas the *k* was 0.0373 and 0.0396 day^−1^ for tetramethrin in sterilized and non-sterilized soil slurries without the strain A16, respectively (Table 3). Therefore, bioaugmentation of tetramethrin-contaminated soils (50 mg·kg^−1^) with strain A16 (1.0 × 10^7^ cells g^−1^ of soil) tremendously accelerated the degradation rate of tetramethrin, and its half-life (*t*_1/2_) was reduced by 125.8 and 110.8 days in sterilized and non-sterilized soil slurries, respectively, in comparison with soil slurries without the strain A16. In addition, no obvious lag period was observed during the degradation experiment in soil slurry with strain A16. The observation of soil slurry suggested that strain A16 has a strong tetramethrin degradation ability in contaminated agricultural fields.

In soil, pyrethroid degradation with microbial strains has been shown to be effective [32,56,57,58]. In the present study, the kinetics of tetramethrin degradation by strain A16 were investigated in a soil environment, indicating that bioremediation of the synthetic pyrethroids was affected by the soil physico-chemical conditions and chemical structure of pyrethroids. Earlier studies also suggested the potential of bacterial genera in soil-based bioaugmentation for effective pyrethroid removal. In one study, permethrin degradation kinetics in soil were investigated, and a significant reduction in half-life in unsterilized and sterilized soil with *Acinetobacter baumanni* strain ZH-14 was reported [23]. Similarly, an effective reduction in the half-life of pyrethroids in soil was reported with *Pseudomonas* and *Bacillus* [21,59,60]. In the present study, we also observed a strong influence of strain A16 in the soil-based bioaugmentation through the removal of tetramethrin.

### 2.7. Metabolite Identification

The results of GC-MS showed that the retention time (RT) of compound A was 26.0 min and the mass/charge ratio (*m*/*z*) was 331.2 (Figure S2). Compound A was identified as tetramethrin according to the fragment retention time, ion fragment characteristics, and the similarity of the corresponding standard compounds in the NIST database. Four intermediate metabolites—B, C, D and E—were detected with the disappearance of tetramethrin residue. The retention times of compounds B, C, D, and E were 23.1, 25.9, 23.5, and 21.3 min, respectively (Figure S2). Based on a comparison with the NIST database, compounds B, C, D, and E were identified as cyclopropylmethanol-2-butyl-4,5,6,7-tetrahydro-1*H*-isoindole-1,3(2*H*)-dione, acrylamide, and *N*-ethylacetamide, respectively. The corresponding compound structure is shown in Table 4. Ion and mass spectra are shown in Figures S3 and S4.

**Figure 8:**
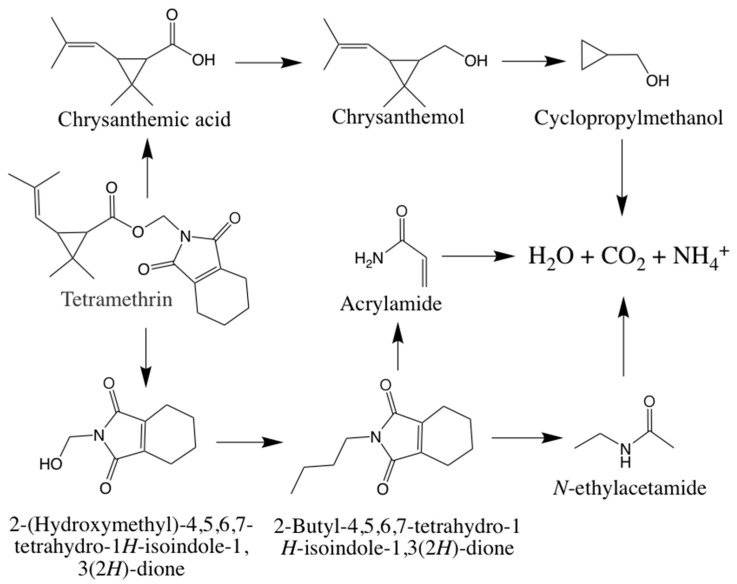
Proposed tetramethrin degradation pathway in strain A16.

Based on the results of metabolite identification of tetramethrin by GC-MS, a novel metabolic pathway for tetramethrin in strain A16 was proposed (Figure 8). Strain A16 first hydrolyzed tetramethrin by cracking the carboxylate bond to produce *N*-(hydroxymethyl)-4,5,6,7-tetrahydro-1*H*-isoindole-1,3(2*H*)-dione and chrysanthemol acid. *N*-(hydroxymethyl)-4,5,6,7-tetrahydro-1*H*-isoindole-1,3(2*H*)-dione was then converted to 2-buty-4,5,6,7-tetrahydro-1*H*-isoindole-1,3(2*H*)-dione. Under the action of A16 hydrolase, 2-buty-4,5,6,7-tetrahydro-1*H*-isoindole-1,3(2*H*)-dione was degraded into acrylamide and *N*-ethylacetamide. At the same time, chrysanthemol acid was transformed into chrysanthemol, which was formed into cyclopropyl methanol. The intermediate products of tetramethrin were eventually reduced to non-toxic small molecules, such as water and carbon dioxide, and this was observed after 11 days of the experiment.

The study of metabolic pathways is crucial to the analysis of the biodegradation potential of microorganisms. Sometimes, the intermediate metabolite is more toxic than the parent compound [61,62,63]. These intermediates are ephemeral, in low concentrations, and are readily converted to smaller molecular compounds by oxidation and hydrolysis [64,65,66]. Microbial degradation of pyrethroids mainly includes hydrolysis and oxidation reactions of ester bonds [67,68,69]. Carboxylesterase is an important type of pyrethroid hydrolase, and is a subtype of esterase. It is capable of hydrolyzing a large number of ester-containing organic compounds [70,71,72,73]. Under the action of carboxylesterase, pyrethroids can produce non-toxic acids and alcohols [74,75,76]. To date, there is scant information about the degradation pathway and intermediate metabolites of tetramethrin in microbes. The intermediate compounds analysis in the present study revealed that strain A16 was able to degrade tetramethrin and remove it completely from the environment. This is the first report of a metabolic pathway of tetramethrin through hydrolysis of the carboxylester bond and cleavage of the five-carbon ring in a microbe, which we propose is of vital importance in the tetramethrin biogeocycle.

## 3. Material and Methods

### 3.1. Chemicals and Media

Tetramethrin (99% purity), D-cyphenothrin (98% purity), chlorempenthrin (94% purity), prallethrin (95% purity), and allethrin (93% purity) were obtained from Wuhan Yuancheng Pharm Co., Ltd. (Wuhan, China). Chromatographic-grade acetone and acetonitrile was obtained from Fisher Scientific (Waltham, MA, USA). All other chemicals and solvents used in this study were of analytical grade. Stock solutions were prepared by dissolving tetramethrin in chromatographic-grade acetone with a concentration of 10 g·L^−1^; these were used for the degradation analysis of tetramethrin, which was stored in a dark bottle in a refrigerator at 4 °C. The mineral salt medium (MSM) had the following composition: 2 g (NH_4_)_2_SO_4_, 0.2 g MgSO_4_·7H_2_O, 0.01 g CaCl_2_·2H_2_O, 0.001 g FeSO_4_·7H_2_O, 1.5 g Na_2_HPO_4_·12H_2_O, and 1.5 g KH_2_PO_4_ per liter of distilled water were used for the cultivation of tetramethrin-degrading soil bacterial strains in this study. The incubation of strain A16 was carried out in Luria Bertani medium (LB) containing 10 g tryptone, 5 g yeast extract and 10 g NaCl per liter, respectively. The pH values of the two media were adjusted to 7.0, then the media were autoclaved at 121 °C for 20 min.

### 3.2. Enrichment, Isolation and Screening of Tetramethrin-Degrading Bacterial Strain A16

Sewage sludge samples were collected from the wastewater treatment tank of a pesticide factory located in Guangzhou, Guangdong Province, China. Enrichment was carried out in MSM to isolate bacteria from obtained soil samples. A total of 5 g of activated sludge sample was added to 100 mL of MSM liquid medium containing tetramethrin stock solution (acetone as solvent), and the final concentration of tetramethrin was 50 mg·L^−1^. After 7 days of incubation at 30 °C and 200 revolutions per minute on a rotary shaker, the concentration of tetramethrin was increased from 50 mg·L^−1^ to 100 mg·L^−1^, 200 mg·L^−1^, 400 mg·L^−1^, and 800 mg·L^−1^, respectively, according to a 5% inoculation amount [56]. Each transfer was cultured for 7 days. Then, the culture medium was transferred four times and spread on LB solid plates at a gradient of 10, 10^2^, 10^3^, and 10^4^ times dilution, and placed in a 30 °C incubator for inverted culture for 2 days. After the LB plate grew out of single colonies, different single colonies were picked for streak purification at least three times. The degradation potential of the isolates was confirmed by high-performance liquid chromatography (HPLC) (Waters, Milford, MA, USA) [3]. One bacterial isolate, designated as A16, exhibited the highest degradation potential, and was therefore selected for further biodegradation studies.

### 3.3. Bacterial Identification

The bacterial strain was characterized and identified by its morphology and genetic analysis based on 16S rDNA gene sequence. The physico-morphological characterization of the bacterial strain was examined by a scanning electron microscope (SEM) (Hitachi, Japan).

Total genomic DNA was extracted with a Master Pure DNA Purification Kit (Epicentre Biotechnologies, Madison, WI, USA) as per the manufacturer instructions. The 16S rDNA gene was PCR-amplified with universal primer pairs, forward primer (27F: 5′-TGACGAGTGGCGGACGGGTG-3′) and reverse primer (1492R: 5′-CCATGGTGTGACGGGCGGTGTG-3′). The 1405 bp 16S rDNA sequences measured by strain A16 were subjected to BLAST comparative analysis in the GenBank Nucleotide Library through a BLAST search in the National Center for Biotechnology Information (NCBI, accessed on 15 July 2020). The related sequences with high homology were selected for multi-sequence alignment. The phylogenetic analysis and phylogenetic tree construction were performed using MAGA X software (Version 10.0) [77].

### 3.4. Growth and Degradation Assays

The bacterial strains were stored in 30% glycerol at −80 °C. Before each experiment, strain A16 was thawed and inoculated in LB medium at 30 °C in a 200 rpm rotary shaker for 24 h. Bacterial cells were harvested by centrifugation (5 min, 4000× *g*) and washed twice with sterile saline (0.9% NaCl) during the late-exponential growth before inoculation. Unless otherwise stated, the densities of strain A16 were adjusted with sterile *N*-saline to approximately 1.0 × 10^7^ colony-forming units (CFU) per milliliter.

For the growth and degradation experiments, triplicate cultures were grown in MSM supplemented with 50 mg·L^−1^ of tetramethrin as the sole carbon source at 30 °C and 200 revolutions per minute on a rotary shaker for 11 days. A non-inoculated culture served as a control. All experiments were carried out in triplicate under the optimal degradation conditions. Samples were collected periodically from the cultures. Growth was monitored by measuring the optical density (OD) value at 600 nm by UV-spectrophotometer (Shimadzu, Japan), and the amount of residual tetramethrin was determined by HPLC as described below.

### 3.5. Different Initial Concentrations of Tetramethrin Degradation

To investigate the effect of different initial concentrations on the degradation of tetramethrin by strain A16, a single-factor test was designed using different concentrations. Samples were set at six concentration gradients (25, 50, 100, 200, 400, and 800 mg·L^−1^). In each concentration group, 1 mL of bacterial suspension was inoculated in MSM medium of each sample. The samples were incubated at 30 °C and 200 revolutions per minute on a rotary shaker for 11 days. The degradation of different concentrations of tetramethrin by strain A16 was determined by HPLC. Each test was performed in triplicate with non-inoculated samples as the control. The residual pesticide concentration was determined by HPLC.

As tetramethrin itself is the growth substrate of strain A16, with the increase of tetramethrin concentration it also plays the role of microbial growth inhibitor. Therefore, the Andrews equation was used to simulate the degradation kinetics of strain A16 at different tetramethrin concentrations [38]. The Andrews equation is as follows:

(2) $$q=\frac{q_{max}S}{S+K_{s}+\left(S^{2}/K_{i}\right)}$$

where *q*_max_ is the maximum specific degradation rate, *K*_s_ denotes the half-saturation point, *S* represents the inhibitor concentration, and *K*_i_ denotes the substrate inhibition constant.

### 3.6. Optimization of Tetramethrin Degradation Conditions

Response surface technology (RSM) is a common method to optimize the growth and degradation conditions of microorganisms. RSM has great advantages, as it requires fewer experimental runs to efficiently estimate quadratic surfaces for the optimization of responses [23]. Therefore, RSM was employed using a Box–Behnken design to optimize the crucial factors and interactive influences which significantly affect tetramethrin degradation by A16; this was performed in triplicate of the center points to estimate experimental error [78]. The significant factors pH, temperature, and inoculum size were selected as independent variables according to the results of preliminary one-factor-at-a-time experiments. The dependent variable was the degradation of 50 mg·L^−1^ tetramethrin in MSM after 11 days. The experiment, which consisted of 15 experimental runs performed in triplicate at the midpoint, was generated using Design Expert software (Table 5). The experimental data from the model experiments using the polynomial regression analysis were used to predict optimum degradation conditions based on the following equation:

*Y*_i_ = *b*_0_ + ∑*b*_i_*X*_i_ + ∑*b*_ij_*X*_i_*X*_j_ + ∑*b*_ii_*X*_i_^2^ (3)

where *Y*_i_ is the predicted response, *X*_i_ and *X*_j_ are variables, *b*_0_ is the constant, *b*_i_ is the linear coefficient, *b*_ij_ is the interaction coefficient, and *b*_ii_ is the quadratic coefficient.

### 3.7. Degradation of Various Substrates by A16

To explore its ability to degrade various synthetic pyrethroids, strain A16 was inoculated into sterilized MSM supplemented with 50 mg·L^−1^ permethrin, bifenthrin, fenvalerate, beta-cypermethrin, allethrin, prallethrin, chlorempenthrin, and D-cyphenothrin. All cultures were conducted in triplicate with a 50 mL conical flask and incubated at the optimum cultural conditions for 11 days with non-inoculated cultures that served as controls. Triplicate cultures were incubated in MSM at 30 °C and 200 revolutions per minute on a rotary shaker. Sampling was carried out at regular intervals for 11 days and the number of residual pesticides was measured by HPLC as described below.

### 3.8. Biodegradation of Tetramethrin in Soil Slurry

The soil slurry method was used to investigate the degradation of tetramethrin in the field by strain A16. Soil samples were collected from the top layer of soil (3–10 cm) to examine the tetramethrin degradation pattern, taken from the agricultural experimental field of South China Agricultural University, Guangdong Province, China. No pesticide was applied for more than five years. The sample soil was placed in a cool ventilated place and naturally dried. After drying, the soil was milled and passed through a 2 mm sieve. A small amount of soil was sterilized at 121 °C for 120 min. About 10 g of autoclaved soil was transferred into a 250 mL flask that contained 50 mL of MSM. Then, 1 mL active bacterial inoculum was inoculated into each flask containing 50 mg·L^−1^ tetramethrin incubated at 30 °C and 200 revolutions per minute on a rotary shaker to yield a final bacterial count of approximately 1.0 × 10^7^ cells g^−1^ of soil. Tetramethrin residues were extracted at 1, 3, 5, 7, 9, and 11 days of culture, and flasks without the bacterial strain served as the control. Extracted tetramethrin was quantified through HPLC.

### 3.9. Identification of Tetramethrin Metabolites

To identify tetramethrin and its metabolic products of biodegradation, strain A16 was grown in MSM media containing 50 mg∙L^−1^ of tetramethrin, and samples were collected from MSM on 1, 3, 5, 7, 9, and 11 days of incubation. Non-inoculated samples containing the same amount of tetramethrin served as the control. Metabolites were detected by gas chromatography–mass spectrometry (GC-MS) (Agilent 6890N/5975, Santa Clara, CA, USA). Specially, the derivatization of metabolites before GC-MS was not needed in this study. To identify the metabolites, mass spectrometry analyses were matched with authentic standard compounds from the National Institute of Standards and Technology (NIST, Gaithersburg, MD, USA) library database.

### 3.10. Kinetic Analysis

To confirm the effect of strain A16 on tetramethrin degradation, a first-order kinetic model was followed to describe the biodegradation process [79]:

(4) $$C_{t}=C_{0}\times e^{-kt}$$

where *C*_0_ is the initial concentration of tetramethrin (mg·L^−1^), *C*_t_ is the content of tetramethrin at time *t*, *k* is the degradation constant (day^−1^), and *t* is the degradation time (days).

Equation (5) was used to calculate the theoretical half-life (*t*_1/2_) of tetramethrin:

*t*_1/2_ = ln 2/*k* (5)

where ln 2 is the natural logarithm of 2 and *k* is the degradation constant.

### 3.11. Chemical Analysis

Tetramethrin and its metabolites were extracted from MSM medium with a similar extraction method, and a 50 mL centrifuge tube was used to replace the traditional separation funnel. A 2 mL sample was added to the 50 mL centrifuge tube along with 10 mL ethyl acetate, and this was vortexed for 2 min. The tube was kept at room temperature until the water phase and organic phase were clearly stratified. Then, 1 mL organic phase was transferred to a 2 mL centrifuge tube, evaporated and dried by rotary evaporator, and recovered by chromatographic acetonitrile. The recovered samples were collected using a 0.22 μm filter membrane and stored in a brown bottle at 4 °C before HPLC detection. Gas chromatography–mass spectrometry (GC-MS) was used to determine the degradation products of tetramethrin.

Tetramethrin was quantified with a Waters 2690 HPLC system equipped with a Phenomenex C_18_ reverse phase column (250 nm × 4.60 mm, 5 µm) and UV detector. The mobile phase was composed of acetonitrile and deionized water (75:25) at a flow rate of 1.0 mL·min^−1^. The injection volume and detection wavelength were 10 μL and 235 nm, respectively [80,81].

The metabolites of tetramethrin were detected by Agilent 5977B/79800B GC-MS. GC-MS analysis was performed on a DB-5MS quartz capillary column (30.0 cm × 250 μm × 0.25 μm). The carrier gas used was helium (99.999% purity) and its flow rate was 1.5 mL·min^−1^. The injection volume was 1 μL without split injection. The full scan range was 30–500 nm to analyze diverse metabolites. The detector MSD ion source temperature was 230 °C; the quadrupole temperature was 150 °C; and the inlet temperature was 250 °C. The initial column temperature was maintained at 90 °C for 2 min, and was then increased to 150 °C incrementally by 6 °C per minute for 1 min. Then, it was raised to 180 °C at an increment of 10 °C per minute and maintained for 4 min. Finally, the temperature was increased to 260 °C by 20 °C per minute for 10 min [41,82].

## 4. Conclusions

The present study identified a novel bacterial isolate *G. cholesterolivorans* A16 with strong tetramethrin degradation activity. Strain A16 utilized tetramethrin as the sole carbon source for its growth over a wide range of temperatures (20–40 °C) and pH values (5–9), indicating that the strain has excellent applicability in different environments. It is noteworthy that strain A16 tolerated and degraded tetramethrin up to a concentration of 800 mg·L^−^^1^, thus giving it an exceptional ability to colonize ecological niches where pesticide concentrations are high. This study first demonstrated that the bacterium had complete detoxification and metabolic pathways of tetramethrin. Moreover, in experiments conducted with soil, strain A16-based bioaugmentation efficiently degraded tetramethrin at a significantly reduced half-life, demonstrating that it has great advantages in bioremediation of tetramethrin-contaminated water and soil due to its adaptability in different environments. Furthermore, strain A16 was capable of degrading a wide range of synthetic pyrethroids, suggesting that it is a potent and effective candidate for the bioremediation of pyrethroid-contaminated terrestrial and aquatic environments. However, further in-depth studies of its interaction with the environment and the degradation genes that encode for key enzymes are needed to develop a safe and efficient strategy to clean up tetramethrin contamination.

## Figures and Tables

**Table 1:** Box–Behnken random design matrix and dependent variable response to tetramethrin degradation.

Run	*X*_1_	*X*_2_	*X*_3_	*Y*
1	5	20	0.6	86.7
2	9	20	0.6	93.6
3	5	40	0.6	90.7
4	9	40	0.6	100
5	5	30	0.3	85.5
6	5	30	0.9	91.5
7	9	30	0.3	100
8	9	30	0.9	100
9	7	20	0.3	88.4
10	7	40	0.3	93.7
11	7	20	0.9	94.2
12	7	40	0.9	100
13	7	30	0.6	97.1
14	7	30	0.6	96.6
15	7	30	0.6	98.5

Note: *X*_1_ = Temperature; *X*_2_ = pH; *X*_3_ = Inoculum size; *Y* = Degradation (%).

**Table 2:** Analysis of variance (ANOVA) of the fitted quadratic polynomial model for tetramethrin degradation.

Source	DF	SS	MS	*F*-Value	*p*-Value *
Model	9	341.1	37.9	15.3	0.0039
*X*_1_	1	194.1	194.1	78.4	0.0003
*X*_2_	1	57.4	57.4	23.2	0.0048
*X*_3_	1	41.0	41.0	16.5	0.0096
*X*_1_ *X*_2_	1	1.3	1.3	0.55	0.4887
*X*_1_ *X*_3_	1	9.0	9.0	3.6	0.1139
*X*_2_ *X*_3_	1	0.06	0.06	0.02	0.8799
*X*_1_^2^	1	18.5	18.5	7.5	0.0408
*X*_2_^2^	1	21.0	21.0	8.5	0.0331
*X*_3_^2^	1	2.9	2.9	1.2	0.3235
Residual	7	12.3	2.4		
Lack of Fit	3	10.3	3.4	3.4	0.2320
Pure Error	4	2	0.99		
Cor Total	16	353.4			

Note: *X*_1_ = Temperature; *X*_2_ = pH; *X*_3_ = Inoculum size. DF refers to degrees of freedom; SS refers to sum of sequences; MS refers to mean square. * *p* level < 0.05 indicates that model terms are significant.

**Table 3:** Kinetic parameters of tetramethrin degradation with A16 in different soil slurries.

Treatments	Regression Equation	*k*	*R* ^2^	*t*_1/2_
Sterilized soil slurry	*C_t_* = 50.6*e*^−0.0048*t*^	0.0048	0.9304	144.4 ± 1.1
Unsterilized soil slurry	*C_t_* = 51.1*e*^−0.0054*t*^	0.0054	0.8425	128.3 ± 0.7
Sterilized soil slurry + A16	*C_t_* = 51.2*e*^−0.037*3t*^	0.0373	0.7004	18.6 ± 1.0
Unsterilized soil slurry + A16	*C_t_* = 50.1*e*^−0.0396*t*^	0.0396	0.7999	17.5 ± 1.5

Note: *k* refers to degradation constant (day^−1^), *t* refers to the degradation time (days), and *R*^2^ refers to determination coefficient. The data presented are means of three replicates with standard deviation.

**Table 4:** Tetramethrin and its intermediate metabolites during degradation with strain A16 in GC-MS analysis.

Code	RT (Minutes)	*m*/*z*	Compound Structure	Chemical Name
A	26.0	331.2	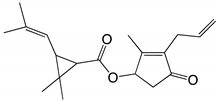	Tetramethrin
B	23.1	154.1	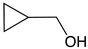	Cyclopropylmethanol
C	25.9	207.1	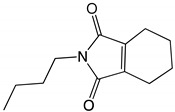	2-Butyl-4,5,6,7-tetrahydro-1*H*-isoindole-1,3(2*H*)-dione
D	23.5	71.0	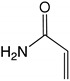	Acrylamide
E	21.3	87.1	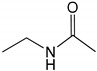	*N*-ethylacetamide

Note: RT refers to retention time (minutes) and *m*/*z* refers to mass/charge ratio.

**Table 5:** The code and levels of three independent variables used in Box–Behnken design.

Independent Variables	Code	Code Levels of Variables		
		−1	0	1
Temperature (°C)	*X*_1_	20	30	40
pH	*X*_2_	5	7	9
Inoculum size (OD_600_)	*X*_3_	0.3	0.6	0.9

## Supplementary Materials

The following are available online at https://www.mdpi.com/article/10.3390/ijms22179242/s1.

## References

[1] Lybrand D.B., Xu H., Last R.L., Pichersky E. (2020). How Plants synthesize pyrethrins: Safe and biodegradable insecticides. Trends. Plant. Sci..

[2] Chrustek A., Hołyńska-Iwan I., Dziembowska I., Bogusiewicz J., Wróblewski M., Cwynar A. (2018). Current research on the safety of pyrethroids used as insecticides. Medicina.

[3] Bhatt P., Huang Y., Rene E.R., Kumar A.J., Chen S. (2020). Mechanism of allethrin biodegradation by a newly isolated *Sphingomonas trueperi* strain CW3 from wastewater sludge. Bioresour. Technol..

[4] Zhang H., Zhang Y., Hou Z., Wang X., Wang J., Lu Z. (2016). Biodegradation potential of deltamethrin by the *Bacillus cereus* strain Y1 in both culture and contaminated soil. Int. Biodeterior. Biodegrad..

[5] Nasuti C. (2003). Different effects of type I and type II pyrethroids on erythrocyte plasma membrane properties and enzymatic activity in rats. Toxicology.

[6] Zhai Y., Li K., Song J., Shi Y., Yan Y. (2012). Molecular cloning, purification and biochemical characterization of a novel pyrethroid-hydrolyzing carboxylesterase gene from *Ochrobactrum anthropi* YZ-1. J. Hazard. Mater..

[7] Yoshida T. (2009). Simultaneous determination of 18 pyrethroids in indoor air by gas chromatography/mass spectrometry. J. Chromatogr. A.

[8] Diegelmann C., Weber J., Heinzel-Wieland R., Kemme M. (2015). Characterization of a cypermethrin-degrading *Methylobacterium* sp. strain A-1 and molecular cloning of its carboxylesterase gene: Cypermethrin biodegradation. J. Basic Microbiol..

[9] Liu H., Hussain S.A., Ali D., Omar S.Y.A., Shaik U., Alghamdi H.A.H. (2020). Induced alteration of rat erythrocyte membrane with effect of pyrethroid based compounds. Saudi J. Biol. Sci..

[10] Köprücü K., Aydın R. (2004). The toxic effects of pyrethroid deltamethrin on the common carp (*Cyprinus carpio* L.) embryos and larvae. Pestic. Biochem. Phys..

[11] Hellinghausen G., Readel E.R., Wahab M.F., Lee J.T., Lopez D.A., Weatherly C.A. (2019). Mass spectrometry-sompatible enantiomeric separations of 100 pesticides using core–shell chiral stationary phases and evaluation of iterative curve fitting models for overlapping peaks. Chromatographia.

[12] García M.Á., Menéndez-López N., Boltes K., Castro-Puyana M., Marina M.L. (2017). A capillary micellar electrokinetic chromatography method for the stereoselective quantitation of bioallethrin in biotic and abiotic samples. J. Chromatogr. A.

[13] Ma Y., Chen L., Lu X., Chu H., Xu C., Liu W. (2009). Enantioselectivity in aquatic toxicity of synthetic pyrethroid insecticide fenvalerate. Ecotoxicol. Environ. Saf..

[14] Ham J., You S., Lim W., Song G. (2020). Bifenthrin impairs the functions of leydig and sertoli cells in mice via mitochondrion-endoplasmic reticulum dysregulation. Environ. Pollut..

[15] Li H., Qiu Y., Yao T., Ma Y., Zhang H., Yang X. (2020). Evaluation of seven chemical pesticides by mixed microbial culture (PCS-1): Degradation ability, microbial community, and medicago sativa phytotoxicity. J. Hazard. Mater..

[16] Bhatt P., Huang Y., Zhan H., Chen S. (2019). Insight Into microbial applications for the biodegradation of pyrethroid insecticides. Front. Microbiol..

[17] Vaze V.K. (2017). Biodegradation of Pesticide Cypermethrin by phosphatase and esterase enzymes produced by actinomycetes. Res. J. Pharm. Technol..

[18] Castro-Gutiérrez V., Fuller E., Thomas J.C., Sinclair C.J., Johnson S., Helgason T. (2020). Genomic basis for pesticide degradation revealed by selection, isolation and characterisation of a library of metaldehyde-degrading strains from soil. Soil Biol. Biochem..

[19] Zhao J., Jia D., Du J., Chi Y., Yao K. (2019). Substrate regulation on co-metabolic degradation of *β*-cypermethrin by *Bacillus licheniformis* B-1. AMB Express.

[20] Cycoń M., Piotrowska-Seget Z. (2016). Pyrethroid-degrading microorganisms and their potential for the bioremediation of contaminated soils: A review. Front. Microbiol..

[21] Gangola S., Sharma A., Bhatt P., Khati P., Chaudhary P. (2018). Presence of esterase and laccase in *Bacillus subtilis* facilitates biodegradation and detoxification of cypermethrin. Sci. Rep..

[22] Wang T., Hu C., Zhang R., Sun A., Li D., Shi X. (2019). Mechanism study of cyfluthrin biodegradation by *Photobacterium ganghwense* with comparative metabolomics. Appl. Microbiol. Biotechnol..

[23] Zhan H., Wang H., Liao L., Feng Y., Fan X., Zhang L. (2018). Kinetics and novel degradation pathway of permethrin in *Acinetobacter baumannii* ZH-14. Front. Microbiol..

[24] Tian J., Long X., Zhang S., Qin Q., Gan L., Tian Y. (2018). Screening cyhalothrin degradation strains from locust epiphytic bacteria and studying *Paracoccus acridae* SCU-M53 cyhalothrin degradation process. Environ. Sci. Pollut. Res. Int..

[25] Suleiman M., Schröder C., Kuhn M., Simon A., Stahl A., Frerichs H. (2019). Microbial biofilm formation and degradation of octocrylene, a UV absorber found in sunscreen. Commun. Biol..

[26] Drzyzga O., Fernández de las Heras L., Morales V., Navarro Llorens J.M., Perera J. (2011). Cholesterol degradation by *Gordonia cholesterolivorans*. Appl. Environ. Microbiol..

[27] Kurniati T.H., Rusmana I., Suryani A., Mubarik N.R. (2016). Degradation of polycyclic aromatic hydrocarbon pyrene by biosurfactant-producing bacteria *Gordonia cholesterolivorans* AMP 10. J. Bio. Bio. Edu..

[28] Chen S., Hu Q.B., Hu M.Y., Luo J.J., Weng Q.F., Lai K.P. (2011). Isolation and characterization of a fungus able to degrade pyrethroids and 3-phenoxybenzaldehyde. Bioresour. Technol..

[29] Birolli W.G., Vacondio B., Alvarenga N., Seleghim M.H.R., Porto A.L.M. (2018). Enantioselective biodegradation of the pyrethroid (±)-lambda-cyhalothrin by marine-derived fungi. Chemosphere.

[30] Zhao J., Chim Y., Liu F., Jia D., Yao K. (2015). Effects of two surfactants and beta-cyclodextrin on beta-cypermethrin degradation by *Bacillus licheniformis* B-1. J. Agric. Food Chem..

[31] Xiao Y., Chen S.H., Gao Y., Hu W., Hu M.Y., Zhong G.H. (2015). Isolation of a novel beta-cypermethrin degrading strain *Bacillus subtilis* BSF01 and its biodegradation pathway. Appl. Microbiol. Biotechnol..

[32] Deng W., Lin D., Yao K., Yuan H., Wang Z., Li J., Zou L., Han X., Zhou K., He L. (2015). Characterization of a novel *β*-cypermethrin-degrading *Aspergillus niger* YAT strain and the biochemical degradation pathway of *β*-cypermethrin. Appl. Microbiol. Biotechnol..

[33] Zhan H., Huang Y., Lin Z., Bhatt P., Chen S. (2020). New insights into the microbial degradation and catalytic mechanism of synthetic pyrethroids. Environ. Res..

[34] Zhao J., Jia D., Chi Y., Yao K. (2020). Co-metabolic enzymes and pathways of 3-phenoxybenzoic acid degradation by *Aspergillus oryzae* M-4. Ecotoxicol. Environ. Saf..

[35] Zhao T., Hu K., Li J., Zhu Y., Liu A., Yao K., Liu S. (2021). Current insights into the microbial degradation for pyrethroids: Strain safety, biochemical pathway, and genetic engineering. Chemosphere.

[36] Bhatt P., Rene E.R., Kumar A.J., Gangola S., Kumar G., Sharma A. (2021). Fipronil degradation kinetics and resource recovery potential of *Bacillus* sp. strain FA4 isolated from a contaminated agricultural field in Uttarakhand, India. Chemosphere.

[37] Birolli W.G., Arai M.S., Nitschke M., Porto A.L.M. (2019). The pyrethroid (±)-lambda-cyhalothrin enantioselective biodegradation by a bacterial consortium. Pestic. Biochem. Phys..

[38] Huang Y., Lin Z., Zhang W., Pang S., Bhatt P., Rene E.R., Kumar A.J., Chen S. (2020). New insights into the microbial degradation of *D*-cyphenothrin in contaminated water/soil environments. Microorganisms.

[39] Liu F., Chi Y., Wu S., Jia D., Yao K. (2014). Simultaneous degradation of cypermethrin and its metabolite, 3-phenoxybenzoic acid, by the cooperation of *Bacillus licheniformis* B-1 and *Sphingomonas* sp. SC-1. J. Agric. Food Chem..

[40] Cycoń M., Mrozik A., Piotrowska-Seget Z. (2017). Bioaugmentation as a strategy for the remediation of pesticide-polluted soil: A review. Chemosphere.

[41] Chen S., Luo J., Hu M., Lai K., Geng P., Huang H. (2013). Enhancement of cypermethrin degradation by a coculture of *Bacillus cereus* ZH-3 and *Streptomyces aureus* HP-S-01. Bioresour. Technol..

[42] Birolli W.G., Lima R.N., Porto A.L.M. (2019). Applications of marine-derived microorganisms and their enzymes in biocatalysis and biotransformation, the underexplored potentials. Front. Microbiol..

[43] Wu P.C., Liu Y.H., Wang Z.Y., Zhang X.Y., Li H., Liang W.Q., Luo N., Hu J.M., Lu J.Q., Luan T.G. (2006). Molecular cloning, purification, and biochemical characterization of a novel pyrethroid-hydrolyzing esterase from *Klebsiella* sp. strain ZD112. J. Agric. Food Chem..

[44] Chen S.H., Luo J.J., Hu M.Y., Geng P., Zhang Y.B. (2012). Microbial detoxification of bifenthrin by a novel yeast and its potential for contaminated soils treatment. PLoS ONE.

[45] Suzuki Y., Yoshida M., Sugano T., Shibata A., Kodaka R., Fujisawa T., Katagi T. (2017). Behavior of cyphenothrin in aquatic environment. J. Pestic. Sci..

[46] Zhang C., Jia L., Wang S.H., Qu J., Xu L.L., Shi H.H., Yan Y.C. (2010). Biodegradation of beta-cypermethrin by two *Serratia* spp. with different cell surface hydrophobicity. Bioresour. Technol..

[47] Bhatt P., Rene E.R., Kumar A.J., Zhang W., Chen S. (2020). Binding interaction of allethrin with esterase: Bioremediation potential and mechanism. Bioresour. Technol..

[48] Birolli W.G., Dos Santos A., Pilau E., Rodrigues-Filho E. (2021). New role for a commercially available bioinsecticide: *Bacillus thuringiensis* Berliner biodegrades the pyrethroid cypermethrin. Environ. Sci. Technol..

[49] Tallur P.N., Megadi V.B., Ninnekar H.Z. (2008). Biodegradation of cypermethrin by *Micrococcus* sp. strain CPN 1. Biodegradation.

[50] Chen S., Chang C., Deng Y., An S., Dong Y.H., Zhou J., Hu M., Zhong G., Zhang L.H. (2014). Fenpropathrin biodegradation pathway in *Bacillus* sp. DG-02 and its potential for bioremediation of pyrethroid-contaminated soils. J. Agric. Food. Chem..

[51] Lin Q.S., Chen S.H., Hu M.Y., Rizwan-ul-Haq M., Yang L., Li H. (2011). Biodegradation of cypermethrin by a newly isolated *actinomycetes* HU-S-01 from wastewater sludge. Int. J. Environ. Sci. Technol..

[52] Wang B.Z., Ma Y., Zhou W.Y., Zheng J.W., He J. (2011). Biodegradation of synthetic pyrethroids by *Ochrobactrum tritici* strain pyd-1. World J. Microbiol. Biotechnol..

[53] Chen S., Geng P., Xiao Y., Hu M. (2012). Bioremediation of β-cypermethrin and 3-phenoxybenzaldehyde contaminated soils using *Streptomyces aureus* HP-S-01. Appl. Microbiol. Biotechnol..

[54] Zhao J., Chi Y., Xu Y., Jia D., Yao K. (2016). Co-metabolic degradation of *β*-cypermethrin and 3-phenoxybenzoic acid by co-culture of *Bacillus licheniformis* B-1 and *Aspergillus oryzae* M-4. PLoS ONE.

[55] Xiao Y., Lu Q., Yi X., Zhong G., Liu J. (2020). Synergistic degradation of pyrethroids by the quorum sensing-regulated carboxylesterase of *Bacillus subtilis* BSF01. Front. Bioeng. Biotechnol..

[56] Cycoń M., Żmijowska A., Piotrowska-Seget Z. (2014). Enhancement of deltamethrin degradation by soil bioaugmentation with two different strains of *Serratia marcescens*. Int. J. Environ. Sci. Technol..

[57] Chen S., Dong Y.H., Chang C., Deng Y., Zhang X.F., Zhong G., Song H., Hu M., Zhang L.H. (2013). Characterization of a novel cyfluthrin-degrading bacterial strain *Brevibacterium aureum* and its biochemical degradation pathway. Bioresour. Technol..

[58] Zhang X.Q., Hao X., Huo S., Lin W., Xia X., Liu K., Duan B. (2019). Isolation and identification of the *Raoultella ornithinolytica*-ZK4 degrading pyrethroid pesticides within soil sediment from an abandoned pesticide plant. Arch. Microbiol..

[59] Zhang C., Wang S., Yan Y. (2011). Isomerization and biodegradation of beta-cypermethrin by *Pseudomonas aeruginosa* CH7 with biosurfactant production. Bioresour. Technol..

[60] Yang J., Feng Y., Zhan H., Liu J., Yang F., Zhang K., Zhang L., Chen S. (2018). Characterization of a pyrethroid-degrading *Pseudomonas fulva* strain P31 and biochemical degradation pathway of D-phenothrin. Front. Microbiol..

[61] Sevilla-Morán B., Calvo L., López-Goti C., Alonso-Prados J.L., Sandín-España P. (2017). Photodegradation behaviour of sethoxydim and its commercial formulation Poast^R^ under environmentally relevant conditions in aqueous media. Study of photoproducts and their toxicity. Chemosphere.

[62] Villaverde J.J., Santín-Montanyá I., Sevilla-Morán B., Alonso-Prados J.L., Sandín-España P. (2018). Assessing the effects of alloxydim phototransformation products by QSAR models and a phototoxicity study. Molecules.

[63] Villaverde J.J., Sevilla-Morán B., Sandín-España P., López-Goti C., Alonso-Prados J.L. (2014). Challenges of biopesticides under the European Regulation (EC) No. 1107/2009: An overview of new trends in residue analysis. Stud. Nat. Prod. Chem..

[64] Mishra S., Zhang W., Lin Z., Pang S., Huang Y., Bhatt P., Chen S. (2020). Carbofuran toxicity and its microbial degradation in contaminated environments. Chemosphere.

[65] Huang Y., Zhang W., Pang S., Chen J., Bhatt P., Mishra S., Chen S. (2021). Insights into the microbial degradation and catalytic mechanisms of chlorpyrifos. Environ. Res..

[66] Bhatt P., Joshi T., Bhatt K., Zhang W., Huang Y., Chen S. (2021). Binding interaction of glyphosate with glyphosate oxidoreductase and C–P lyase: Molecular docking and molecular dynamics simulation studies. J. Hazard. Mater..

[67] Tang A., Wang B., Liu Y., Li Q., Tong Z., Wei Y. (2015). Biodegradation and extracellular enzymatic activities of *Pseudomonas aeruginosa* strain GF31 on *β*-cypermethrin. Environ. Sci. Pollut. Res. Int..

[68] Liu X., Liang M., Liu Y., Fan X. (2017). Directed evolution and secretory expression of a pyrethroid-hydrolyzing esterase with enhanced catalytic activity and thermostability. Microb. Cell Factories.

[69] Bhatt P., Bhandari G., Bhatt K., Maithani D., Mishra S., Gangola S., Bhatt R., Huang Y., Chen S. (2021). Plasmid-mediated catabolism for the removal of xenobiotics from the environment. J. Hazard. Mater..

[70] Maloney S.E., Maule A., Smith A.R. (1988). Microbial transformation of the pyrethroid insecticides: Permethrin, deltamethrin, fastac, fenvalerate, and fluvalinate. Appl. Environ. Microb..

[71] Bhatt P., Bhatt K., Sharma A., Zhang W., Mishra S., Chen S. (2021). Biotechnological basis of microbial consortia for the removal of pesticides from the environment. Crit. Rev. Biotechnol..

[72] Wang B., Guo P., Hang B., Li L., He J., Wang B., Li S.P. (2009). Cloning of a novel pyrethroid-hydrolyzing carboxylesterase gene from *Sphingobium* sp. strain JZ-1. Appl. Environ. Microbiol..

[73] Bhatt P., Bhatt K., Huang Y., Lin Z., Chen S. (2020). Esterase is a powerful tool for the biodegradation of pyrethroid insecticides. Chemosphere.

[74] Cao Y., Navarro A.I., Perrella L., Cedergreen N. (2021). Can organophosphates and carbamates cause synergism by inhibiting esterases responsible for biotransformation of pyrethroids?. Environ. Sci. Technol..

[75] Bhatt P., Zhou X., Huang Y., Zhang W., Chen S. (2021). Characterization of the role of esterases in the biodegradation of organophosphate, carbamate, and pyrethroid pesticides. J. Hazard. Mater..

[76] Xu D., Gao Y., Sun B., Ran T., Zeng L., He J., He J., Wang W. (2020). Pyrethroid carboxylesterase PytH from *Sphingobium faniae* JZ-2: Structure and catalytic mechanism. Appl. Environ. Microbiol..

[77] Kumar S., Stecher G., Li M., Knyaz C., Tamura K. (2018). MEGA X: Molecular evolutionary genetics analysis across computing platforms. Mol. Biol. Evol..

[78] Feng Y., Zhang W., Pang S., Lin Z., Zhang Y., Huang Y., Bhatt P., Chen S. (2020). Kinetics and new mechanism of azoxystrobin biodegradation by an *Ochrobactrum anthropi* strain SH14. Microorganisms.

[79] Bhatt P., Huang Y., Zhang W., Sharma A., Chen S. (2020). Enhanced cypermethrin degradation kinetics and metabolic pathway in *Bacillus thuringiensis* strain SG4. Microorganisms.

[80] Birolli W.G., Alvarenga N., Seleghim M.H.R., Porto A.L.M. (2016). Biodegradation of the pyrethroid pesticide esfenvalerate by marine-derived fungi. Mar. Biotechnol..

[81] Bhatt P., Zhang W., Lin Z., Pang S., Huang Y., Chen S. (2020). Biodegradation of allethrin by a novel fungus *Fusarium proliferatum* strain CF2, isolated from contaminated soils. Microorganims.

[82] Aiello F., Simons M.G., van Velde J.W., Dani P. (2021). New insights into the degradation path of deltamethrin. Molecules.

