# Supplementary material for: Novel Mechanism and Kinetics of Tetramethrin Degradation Using an Indigenous Gordonia cholesterolivorans A16
Source: Int J Mol Sci. 2021 Aug 26;22(17):9242. doi: 10.3390/ijms22179242 (PMC8431606; doi:10.3390/ijms22179242)
Supplement: Supplementary file 1 [file ijms-22-09242-s001.zip › ijms-1325090-supplementary.pdf]

Supplementary Materials

Novel mechanism and kinetics of tetramethrin degradation using an indigenous *Gordonia cholesterolivorans* A16

Yuxin Guo[1,2#], Yaohua Huang[1,2#], Shimei Pang[1,2#], Tianhao Zhou[1,2#], Ziqiu Lin[1,2], Hongxiao Yu, Guorui Zhang, Pankaj Bhatt[1,2*], Shaohua Chen[1,2*]

[1]*State Key Laboratory for Conservation and Utilization of Subtropical Agro-bioresources, Integrative Microbiology Research Centre, South China Agricultural University, Guangzhou 510642, China*;

[2]*Guangdong Laboratory for Lingnan Modern Agriculture, Guangzhou 510642, China*

# These authors contributed equally to this work.

[*]**Corresponding authors**

Shaohua Chen, Pankaj Bhatt

South China Agricultural University, Guangzhou 510642, China

Tel: +86-20-8528 8229

Fax: +86-20-8528 0292

Email: shchen@scau.edu.cn (S.C); pankajbhatt.bhatt472@gmail.com (P.B)

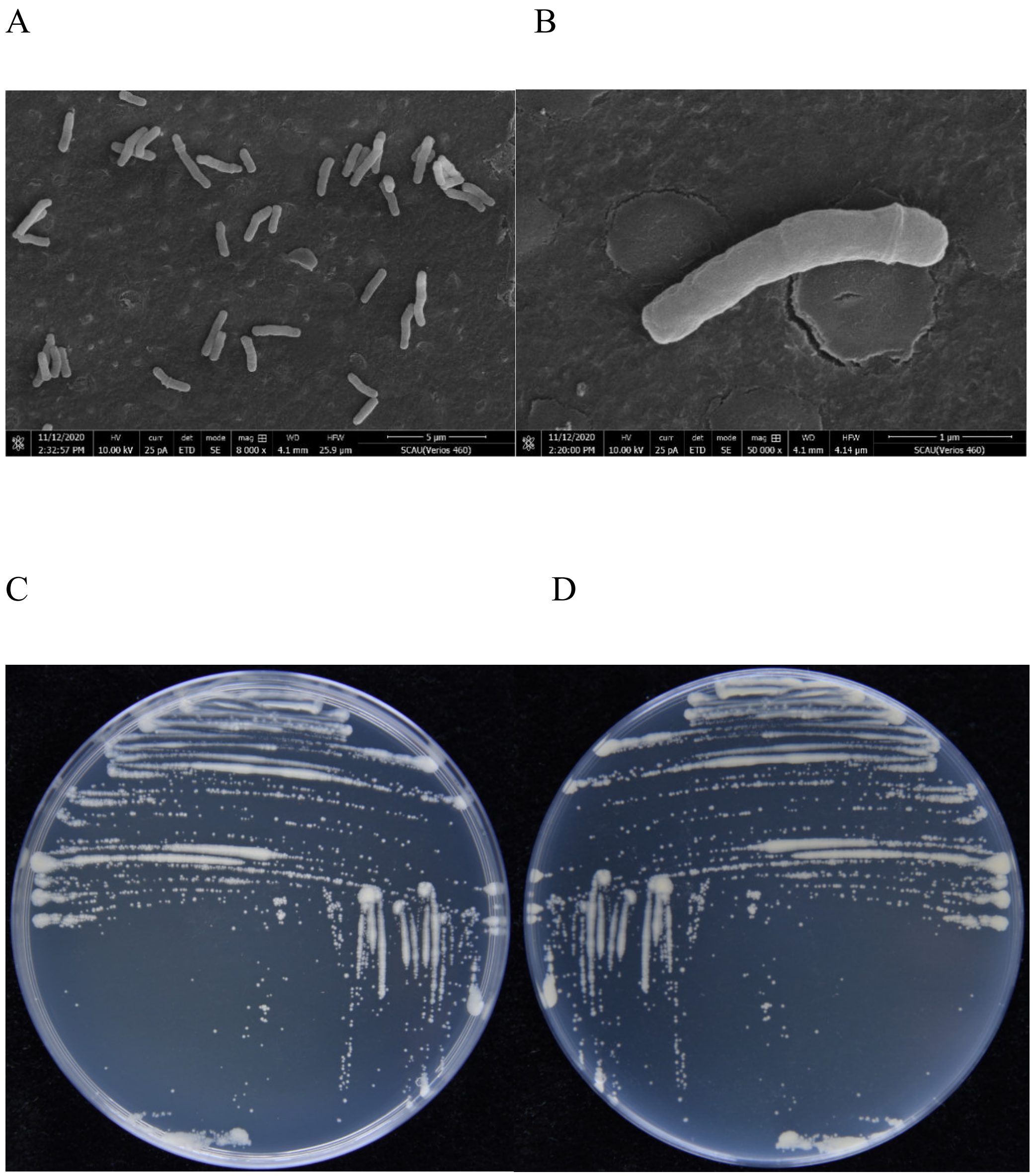

Figure S1. Morphological characteristics of *Gordonia cholesterolivorans* strain A16. Pictures A (1000×) and B (5000×) are scanning electron micrographs. Pictures C and D are front and back sides of strain A16 grown on LB agar plates.

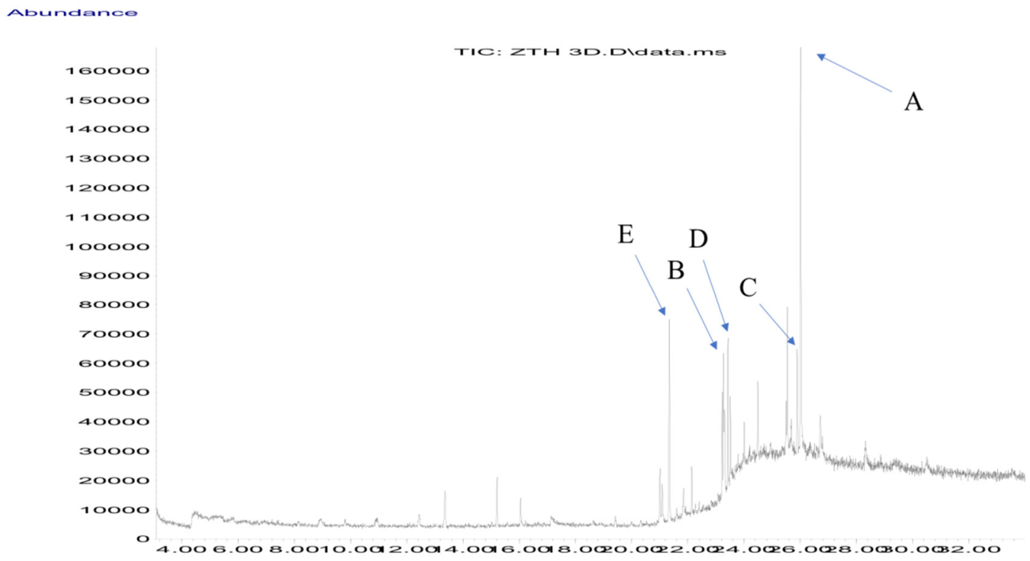

Figure S2. Chromatogram of tetramethrin metabolism by *Gordonia cholesterolivorans* strain A16. (A) Tetramethrin, 26.0 min; (B) cyclopropylmethanol, 23.1 min; (C) 2-butyl-4,5,6,7-tetrahydro-1H-isoindole-1,3(2H)-dione, 25.9 min; (D) acrylamide, 23.5 min; (E) *N*-ethylacetamide, 21.3 min.

A

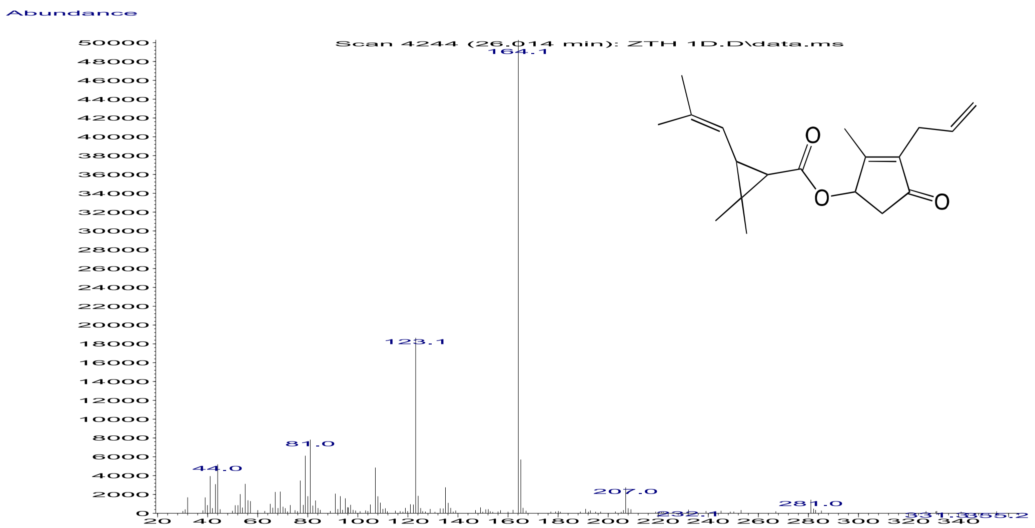
Abundance
Scan 4244 (26.014 min): ZTH 1D.D\data.ms
164.1
123.1
81.0
44.0
207.0
281.0
O
O
O

B

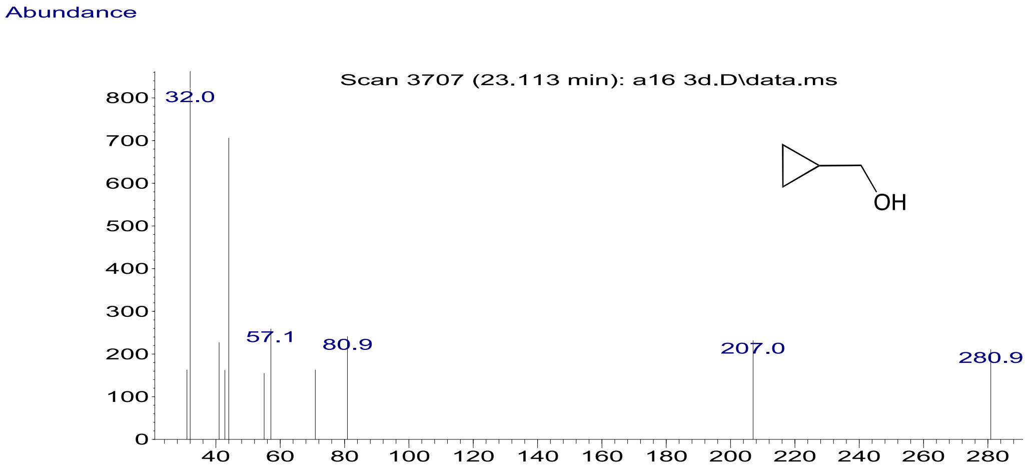
Abundance
Scan 3707 (23.113 min): a16 3d.D\data.ms
32.0
57.1
80.9
207.0
280.9
OH

C

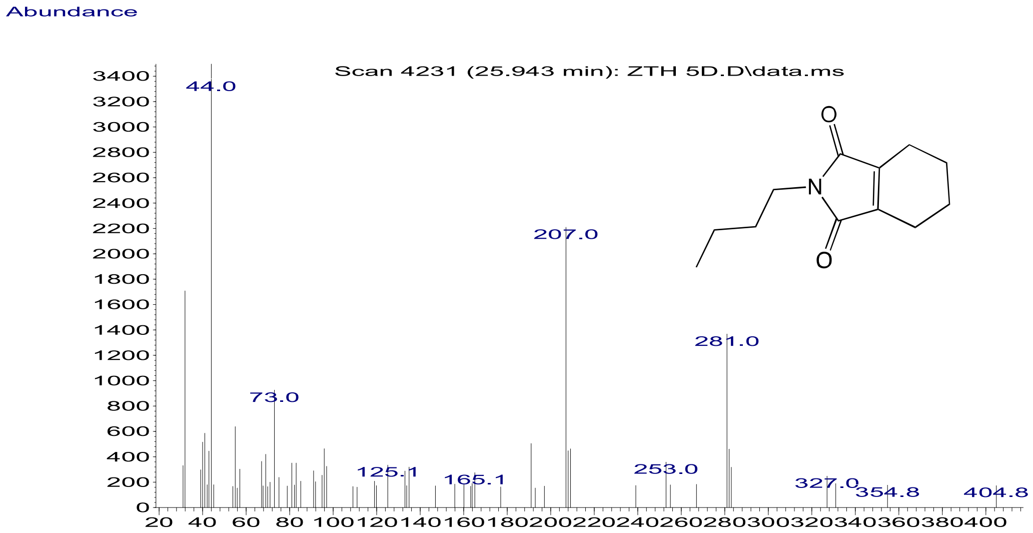
Abundance
Scan 4231 (25.943 min): ZTH 5D.D\data.ms
44.0
73.0
125.1
165.1
207.0
253.0
281.0
327.0
354.8
404.8
O
N
O

D

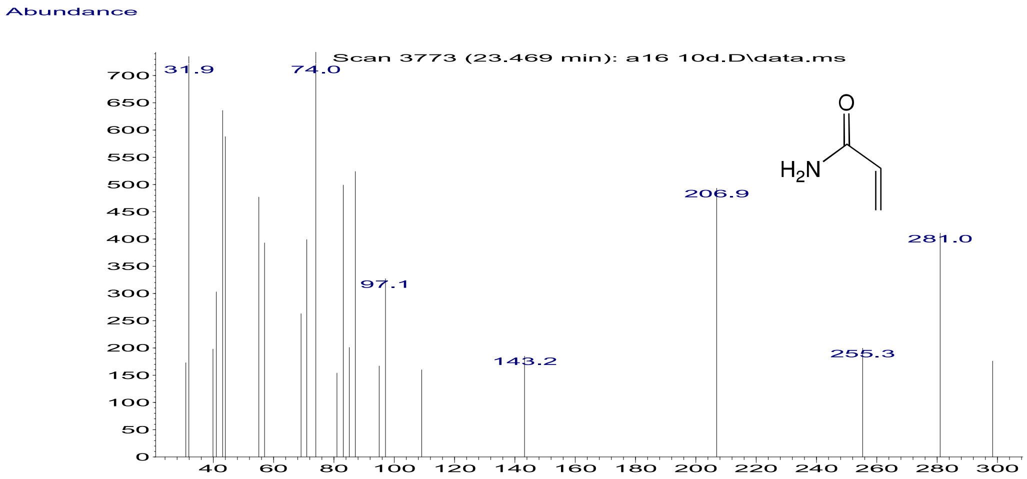
Abundance
Scan 3773 (23.469 min): a16 10d.D\data.ms
31.9
74.0
97.1
143.2
206.9
255.3
281.0
O
H_2N

E

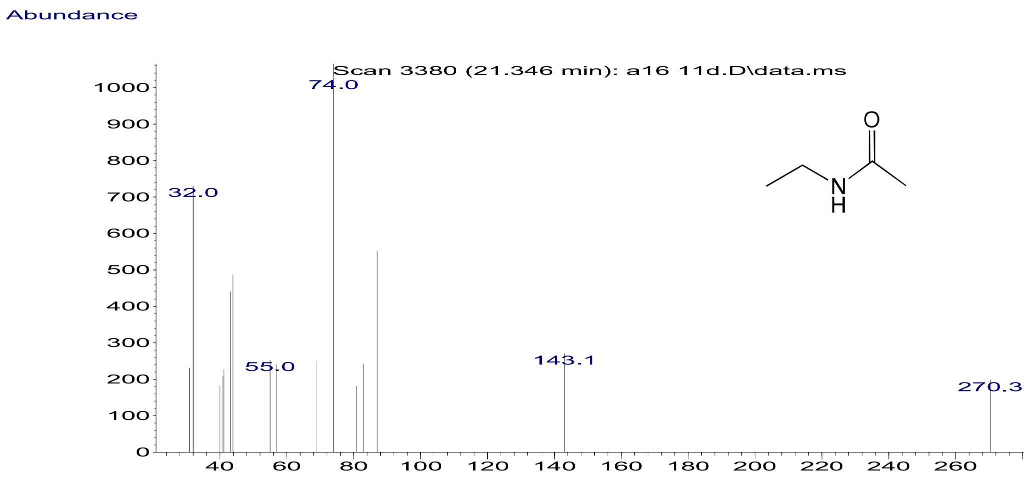

Figure S3. Gas chromatography-mass spectrometry (GC-MS) analysis of the metabolites produced during tetramethrin degradation by *Gordonia cholesterolivorans* strain A16. (A) Tetramethrin; (B) cyclopropylmethanol; (C) 2-butyl-4,5,6,7-tetrahydro-1H-isoindole-1,3(2H)-dione; (D) acrylamide; (E) *N*-ethylacetamide.

A

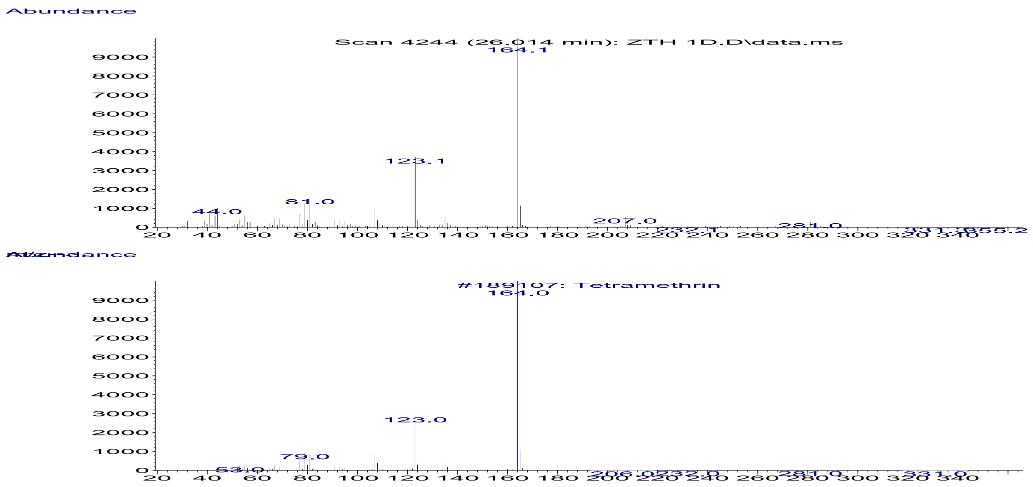
Abundance
Scan 4244 (26.014 min): ZTH 1D.D\data.ms
164.1
123.1
81.0
44.0
207.0
281.0
Abundance
#189107: Tetramethrin
164.0
123.0
79.0
53.0

B

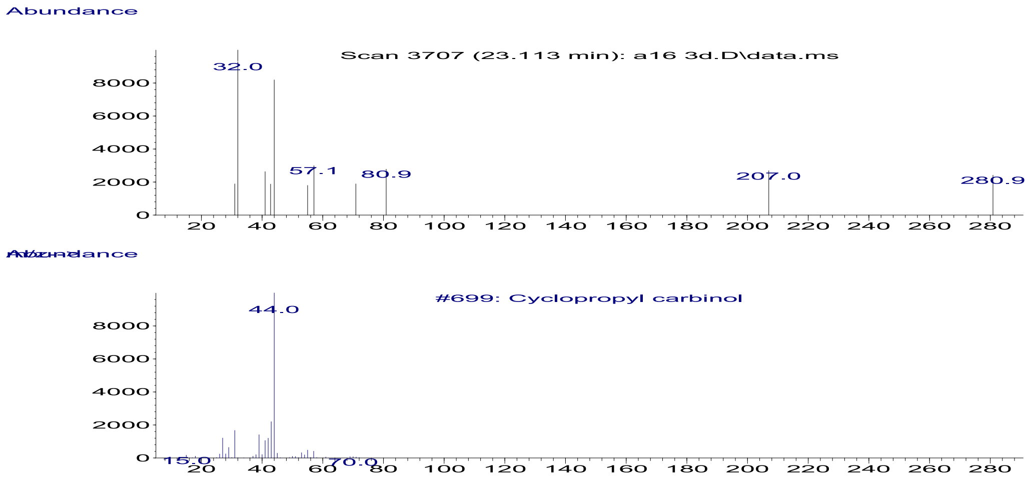
Abundance
Scan 3707 (23.113 min): a16 3d.D\data.ms
32.0
57.1
80.9
207.0
280.9
Abundance
#699: Cyclopropyl carbinol
44.0
15.0
70.0

C

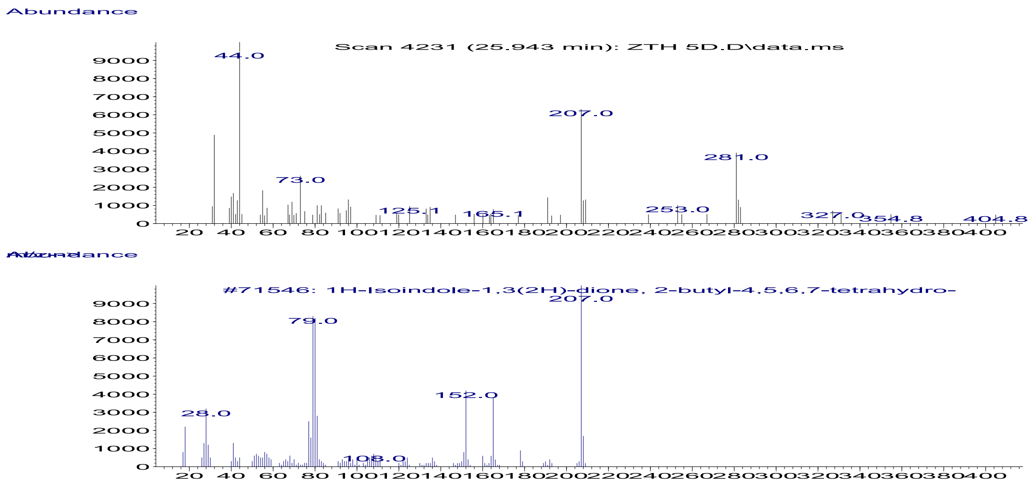
Abundance
Scan 4231 (25.943 min): ZTH 5D.D\data.ms
44.0
207.0
281.0
73.0
125.1
165.1
253.0
327.0
354.8
404.8
#71546: 1H-Isoindole-1,3(2H)-dione, 2-butyl-4,5,6,7-tetrahydro-
207.0
79.0
152.0
28.0
108.0

D

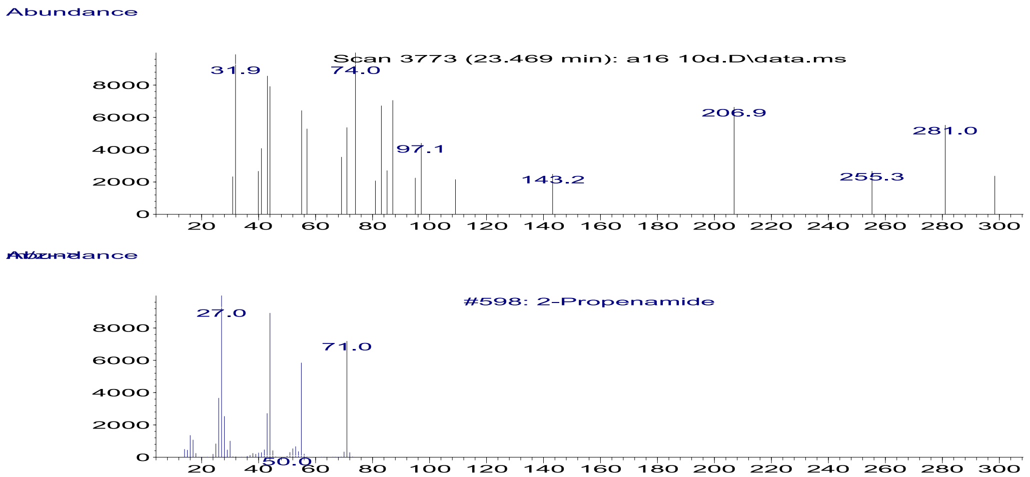
Abundance
Scan 3773 (23.469 min): a16 10d.D\data.ms
31.9
74.0
206.9
281.0
97.1
143.2
255.3
#598: 2-Propenamide
27.0
71.0
50.0

E

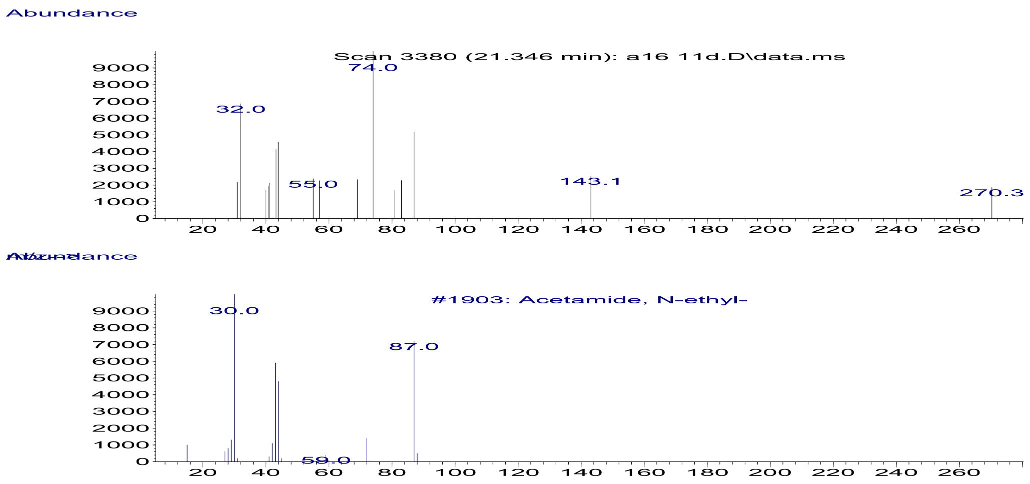

Figure S4. The mass spectra of tetramethrin metabolites reported in the National Institute of Standards and Technology (NIST, United States) library database. (A) Tetramethrin; (B) cyclopropylmethanol; (C) 2-butyl-4,5,6,7-tetrahydro-1H-isoindole-1,3(2H)-dione; (D) acrylamide; (E) *N*-ethylacetamide.
